# Does treadmill workstation use affect user’s kinematic gait symmetry?

**DOI:** 10.1371/journal.pone.0261140

**Published:** 2021-12-14

**Authors:** Paul Gonzalo Arauz, María-Gabriela García, Mauricio Velez, Cesar León, Francisco Velez, Bernard Martin

**Affiliations:** 1 Department of Mechanical Engineering, Universidad San Francisco de Quito, Quito, Pichincha, Ecuador; 2 Department of Industrial Engineering, Universidad San Francisco de Quito, Quito, Pichincha, Ecuador; 3 Department of Industrial and Operations Engineering, University of Michigan, Ann Arbor, Michigan, United States of America; University of Massachusetts Lowell, UNITED STATES

## Abstract

The effects of treadmill workstation use on kinematic gait symmetry and computer work performance remain unclear. The purpose of this pilot study was to analyze the effects of treadmill workstation use on lower body motion symmetry while performing a typing task when compared to overground and treadmill walking. The lower body motion of ten healthy adults (6 males and 4 females) was recorded by a motion capture system. Hip, knee, and ankle joint rotations were computed and compared for each condition. Despite comparable lower body kinematic gait asymmetries across conditions, asymmetric knee flexion motions at early gait cycle were only found in treadmill workstation users (left knee significantly more flexed than the right one). This demonstrates that the interaction between walking and another task is dependent on the task cognitive content. Our findings suggest that lower body kinematic gait symmetry may be influenced by the use of treadmill workstations.

## Introduction

The World Health Organization classified physical inactivity as the fourth leading risk factor accounting for 6% of global mortality [1]. More than 30% of people older than 15 years are physically inactive, with the highest proportion in the United States [2]. Sedentary lifestyle has been associated with cardiometabolic risk, type 2 diabetes and premature mortality [3]. Currently, sedentary behavior has increased in many aspects of life including at home, in commuting, and at the workplace. Wennman et al. [4] showed that the time sitting at a computer has increased in the past few years in different age groups. In many workplaces workers spend most of their time sitting long hours at the computer. Prolonged occupational sitting is associated with a higher risk of musculoskeletal discomfort [5,6], low back pain [7–9]—although controversial [10]—, and a negative impact on cardiometabolic biomarkers [11,12]. Moreover, prolonged occupational sitting has been connected to overweight and obesity [13], which may lead to cardiovascular problems.

Recently, reducing sedentary behavior and increasing physical activity at work has gained attention [14] and the use of dynamic workstations in office work has become more popular [14–17]. Studies on dynamic workstations, such as treadmill workstations, have presented physiological benefits such as increase in daily physical activity, [18], energy expenditure [19–21], reduction of abdominal circumference and cholesterol [18,22], minor increase in walking time of an overweight population [23], among others, [16,24,25]. However, cardiovascular benefits are not demonstrated [26]. Qualitative analyses have also showed that walking while working results in higher work satisfaction, less boredom, and less stress compared to working seated [27,28]. In addition, while studies have indicated that cognitive tasks are not impaired [19,29,30] or general performance is improved [15] when using a treadmill workstation, others found opposing results [31,32]. Few studies have focused on the biomechanical aspects of treadmill workstations use during computer work [33–35]. It was noted that treadmill workstation users shorten their stride length and decrease their base of support when performing mouse-specific tasks [34] or while reading [35]; while Botter et al. [33] concluded that posture is not affected by treadmill workstation use. Furthermore, a classical method known as dual-task paradigm has been employed to address the interrelation between walking and cognition [36]. Recent studies on a dual-task paradigm examining gait asymmetries in able-bodied individuals found that gait asymmetries arise under specific constraints in healthy people as an adaptation to task requirements [36,37]. In addition, asymmetrical behavior of the lower limbs during able-bodied ambulation was found to reflect natural functional differences between the lower extremities [38]. Thus, it is reasonable to hypothesize that treadmill workstation users would display interlimb asymmetries.

Although most current research on gait symmetry analyzes differences in spatiotemporal measures (stance time, swing time, stride length), several studies have utilized kinematic gait symmetry as an effective tool to asses functionality after total hip and knee replacement [39–41]. However, there is currently a paucity of data regarding the potential gait asymmetries in lower body kinematics in treadmill workstation users and the eventual interactions with cognitive tasks. For instance, it is not clear how natural gait movement patterns may be affected while using a treadmill workstation. The eventual alteration of kinematic gait symmetries may contribute to risks associated with discomfort, ailments, and potential injuries. Therefore, the aim of this pilot study is exploratory in nature. Its results will determine whether alteration of lower limb symmetry during treadmill workstation use when compared to overground and treadmill walking may be significant enough to eventually induce injuries or pain.

## Methods

### Participants

Ten healthy adults (6 males, 4 females) were included in this study. All participants were between 18 and 60 years old, presented a body mass index (BMI) inferior to 32 kg/m^2^, and reported a healthy lifestyle (exercised at least twice a week), and with no known gait impairments, disabilities, prior history of injuries that required surgery, auto-immune disease, or cognitive impairment. Nine out of the ten participants were self-determined right-limb dominant, and a 57-year-old participant worked as a fitness trainer. Participant’s anthropometric characteristics were as follows: average age was 25.8±10.6 (range: 20.0–57.0) years, with a stature of 1.7±0.1 (range: 1.6–1.8) m and weight 65.4±.15.1 (range: 42–86) kg, respectively. The average BMI was 21.5±4.3 (range: 16.8–30.1) kg/m^2^. The study protocol was approved the Ethics Committee of the Universidad San Francisco de Quito. Written informed consent approved by the Ethics Committee was obtained for each participant prior to data collection. The study complied with the tenets of the Declaration of Helsinki.

### Instrumentation

**Fig 1:**
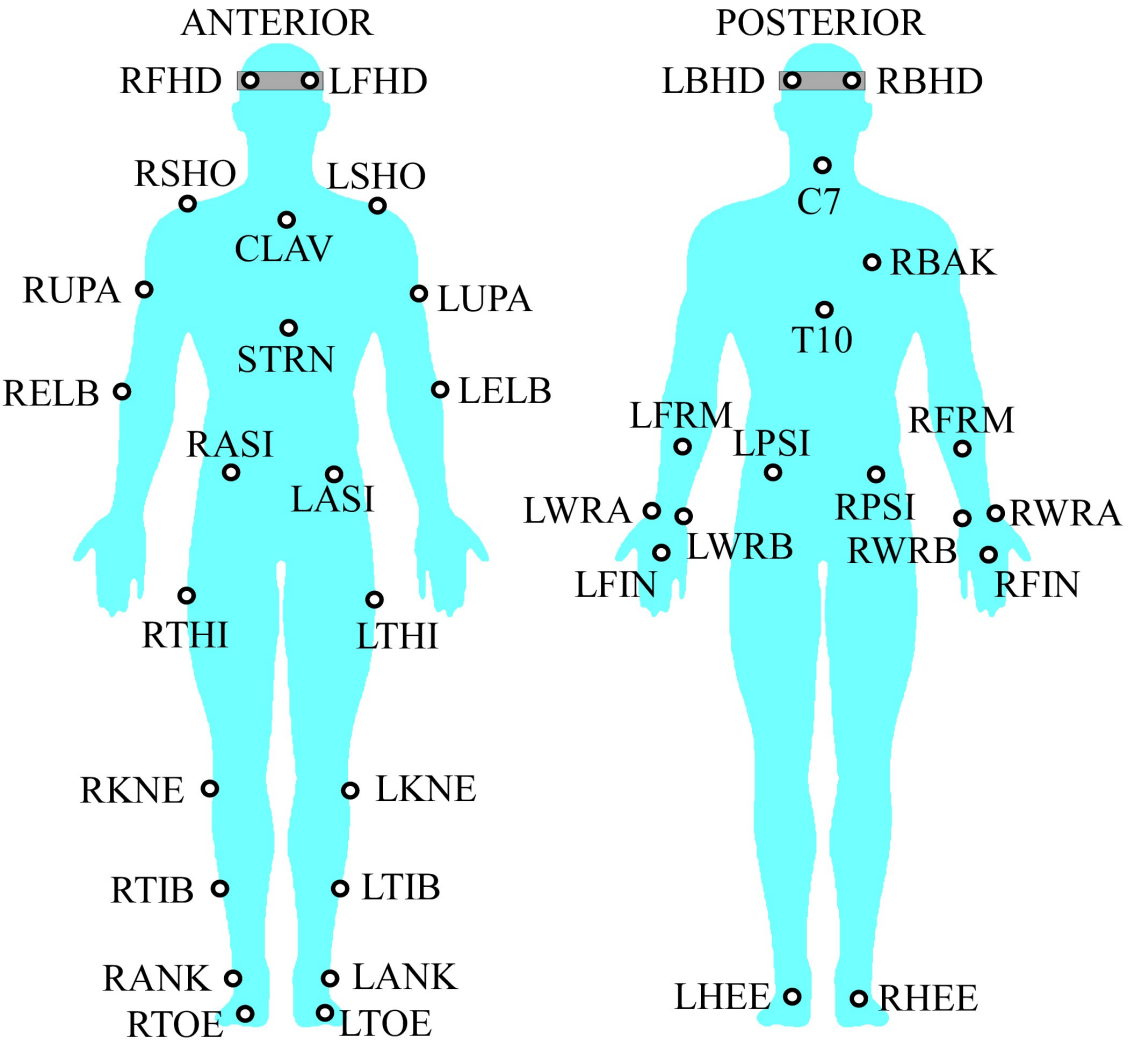
Full body marker set. Prefixes denote the following: L: Left, and R: Right. The following landmarks were used: Suprasternal notch (CLAV), xiphoid process (STRN), spinous process at C7 (C7), spinous process at T10 (T10), acromial angle (BAK), acromioclavicular joint (SHO), upper arm (UPA), forearm (FRM), lateral epicondyle of humerus (ELB), radial styloid (WRA), ulnar stylloid (WRB), third metacarpal (FIN), temple (FHD), back head (BHD), anterior superior iliac spine (ASI), posterior superior iliac spine (PSI), femur (THI), lateral epicondyle of femur (KNE), tibia, (TIB), lateral malleoli (ANK), and distal interphalangeal joint of the first toe (TOE).

A single belt treadmill and a desktop computer were used to evaluate gait movements during a typing task. The workstation height was adjusted to each participant’s anthropometric dimensions to obtain the recommended standard elbow height [35]. A 10-camera motion capture system (Vicon MX, Oxford, UK) connected to a computer running Vicon Nexus software (version 2.10) was used to record motion data at 100 Hz. The cameras space encompassed a volume of 5 x 4 x 4 cubic meters with an accuracy of 0.5 mm, as recommended [42–44]. Thirty-nine reflective spherical markers (∅ 10 mm) were attached to each participant’s anatomical landmarks (Plug-In Gait Marker Set, Vicon MX, Oxford UK) with double-sided tape [42–44], as illustrated in Fig 1.

### Procedures

A power analysis based on the study by Grindle et al. [35], who described the effects of walking workstations on biomechanical performance in nine participants indicated that the projected sample size needed is approximately 10 participants with an alpha = 0.05, power = 0.8, and a sample ratio = 1. Four conditions corresponding respectively to overground walking, treadmill walking, as well as a computer typing task while walking on a treadmill workstation or standing on the still treadmill were tested in a random order. Participants were exposed to all experimental conditions in a single session. Each walking condition was repeated three times consecutively. Each trial included at least five complete gait cycles at a self-selected comfortable speed. Hence, each walking condition, in total, presented at least 15 complete gait cycles and were selected for analyses. Prior to data collection, each participant practiced for 5-minute on the treadmill workstation. This workstation consisted of a single belt commercial treadmill straddled by an adjustable sit-stand desk equipped with a computer including a standard keyboard, a monitor, and a mouse, centered relative to the middle plane of the desk. The desk was adjusted to each participant elbow height, with standard positioning of the video monitor. Even though participants rested their elbows on the desktop to perform the typing task while walking on the treadmill, their hands and elbows moved freely during overground and treadmill walking. Typing is a cognitive load that requires explicit memory for the trained material [45]. Thus, this task was used as a measure of cognition in the present study. Typing accuracy (correct words) and speed (word-per-minute) were evaluated and recorded using the Mavis Beacon^TM^ teaching typing test. Participants completed independent similar-level teaching typing tests during treadmill workstation use and standing conditions.

### Data processing

To analyze gait kinematics, the *x*, *y*, and *z* global coordinates of each marker relative to the Vicon coordinate system were used to compute joint rotation angles of the hip (between pelvis and femur), knee (between femur and tibia), and ankle (between foot and tibia) in the sagittal and frontal planes. Joint rotations were quantified following the recommendation of the International Society of Biomechanics [46] using a Cardan angle sequence (flexion/extension, adduction/abduction) [47]. Data were exported and processed in MATLAB (MathWorks, Inc., Natick, MA) using a custom program. Left and right limb gait kinematic measurements were compared for each condition. The joint angular data was split into individual strides, and a time normalized waveform (0–100%) of the average gait cycle for each condition was generated with 1% sample steps [39,40], with 0% corresponding to heel contact of the concerned leg. Heel-contact and toe-off events were detected using the local maxima in the anterior-posterior position of the heel marker. The rotation angles of the hip, knee, and ankle joints were calculated to evaluate lower limb kinematic gait symmetry in each condition.

### Statistical analysis

Statistical parametric mapping (SPM) [48–50] analyses were performed using scalar fields to determine if there is a significant difference in hip, knee, and ankle motion during gait by comparing joint angle kinematics throughout gait cycle between the left and right sides in each condition. A Student’s t test compared typing precision and speed scores between walking and standing on a treadmill. The statistical analysis was performed in MATLAB (MathWorks, Inc., Natick, MA). The level of significance was set at *p* = 0.05.

## Results

### Walking speed

**Fig 2:**
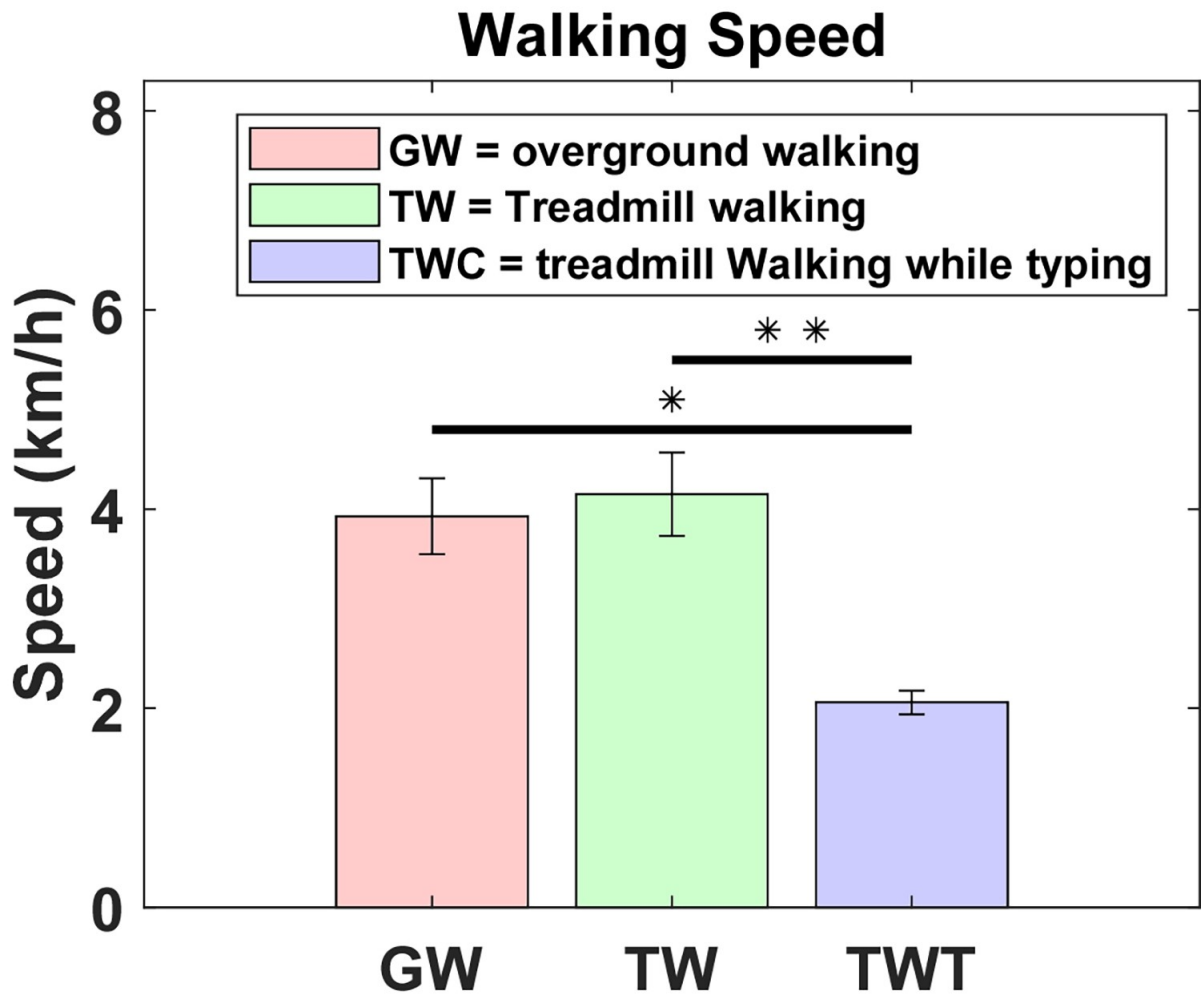
Average and standard deviation walking speed (km/h) during overground walking (GW), treadmill walking (TW), and treadmill walking while typing (TWT), in ten healthy participants. Kruskal-Wallis H tests with pairwise comparisons were performed. * indicates *p* = 0.002 and ** indicates *p*<0.001.

The average overground walking speed was 3.93 km/h ± 0.38 (range: 3.45–4.81). This did not differ significantly (*p* = 0.319) from the average walking speed on the treadmill, which was 4.15 km/h ± 0.42 (range: 3.54–4.83). However, the walking speed was about 50% slower, 2.06 km/h ± 0.12 (range: 1.89–2.2, Fig 2), when walking while typing (*p* = 0.002 and p <0.001, respectively).

### Typing performance

Although the average typing speed score was significantly lower (*p* = 0.006) when walking while typing than while standing still on the treadmill (36.4 ± 9.8, range: 26–59 vs. 40.4 ± 11.7, range: 28–67), no significant differences were found in computer typing accuracy scores between these two conditions (94.6 ± 1.7, range: 92–97 vs. 95.0±2.1, range: 91–98).

### Asymmetric gait motions of lower limb joints

Lower limb gait asymmetric kinematics of hip, knee, and ankle joints were assessed by comparing left and right sides during one gait cycle of overground walking (GW), treadmill walking (TW), and treadmill walking while typing (TWT), in ten healthy participants using 1D SPM analysis.

#### Asymmetric flexion-extension motions

**Fig 3:**
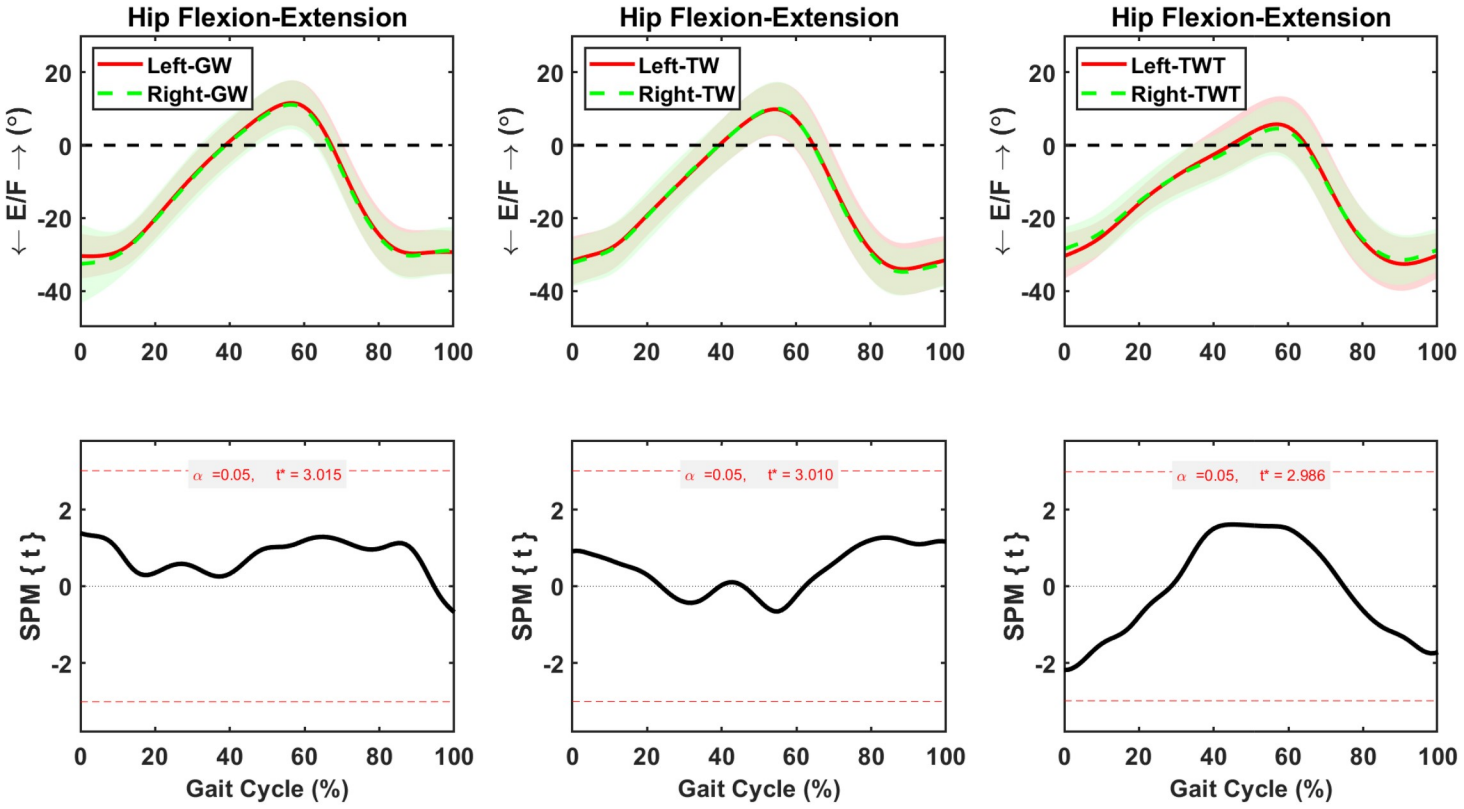
Average and standard deviation of hip extension/flexion (E/F) for left and right sides during one gait cycle of overground walking (GW), treadmill walking (TW), and treadmill walking while typing (TWT), in ten healthy participants. Scalar field SPM results with threshold at *t* >3.0 depicting where, in % cycle, left side angles were greater and lesser than right side angles. In upper panel: Solid and dashed lines correspond to average left and right sides, and shaded areas correspond to standard deviation.

**Fig 4:**
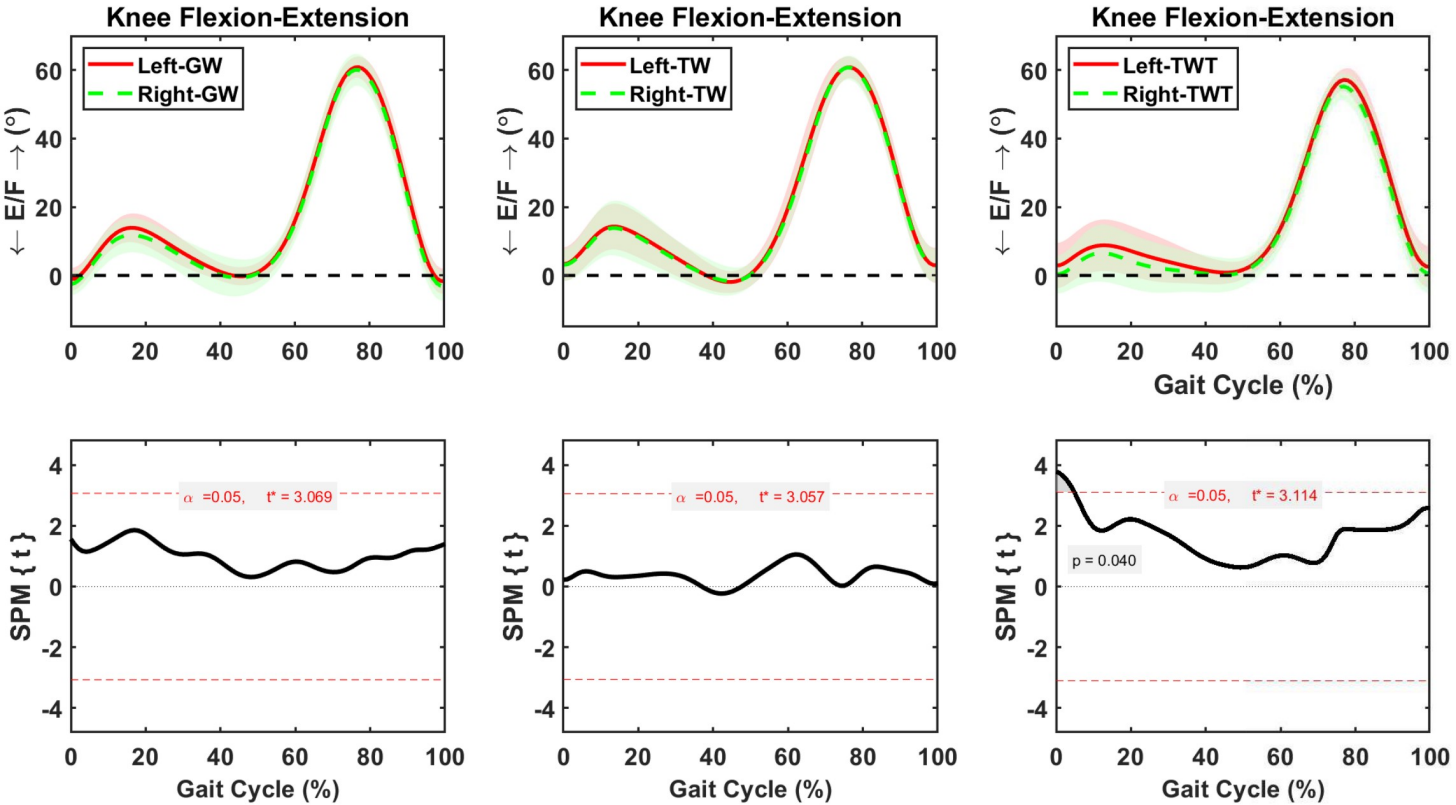
Average and standard deviation of knee extension/flexion (E/F) for left and right sides during one gait cycle of overground walking (GW), treadmill walking (TW), and treadmill walking while typing (TWT), in ten healthy participants. Scalar field SPM results with threshold at *t* >3.0 depicting where, in % cycle, left side angles were greater and lesser than right side angles. In upper panel: Solid and dashed lines correspond to average left and right sides, and shaded areas correspond to standard deviation.

**Fig 5:**
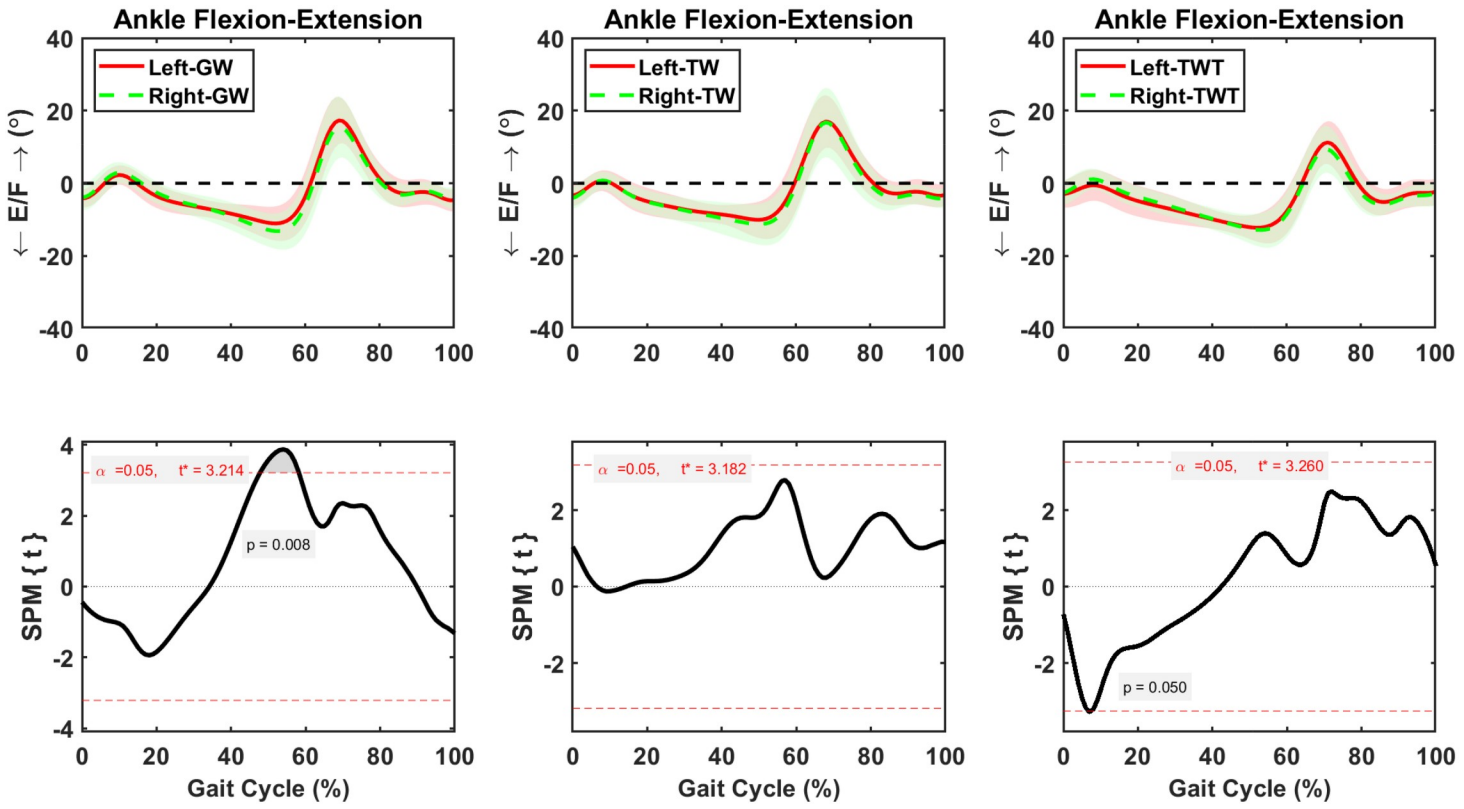
Average and standard deviation of ankle extension/flexion (E/F) for left and right sides during one gait cycle of overground walking (GW), treadmill walking (TW), and treadmill walking while typing (TWT), in ten healthy participants. Scalar field SPM results with threshold at *t* >3.0 depicting where, in % cycle, left side angles were greater and lesser than right side angles. In upper panel: Solid and dashed lines correspond to average left and right sides, and shaded areas correspond to standard deviation.

Hip flexion-extension was symmetric between sides during the whole gait cycle in GW, TW, and TWT conditions (Fig 3). Whereas knee flexion-extension remained symmetric between sides during the whole gait cycle in GW and TW conditions (Fig 4), significantly higher flexion (*p* = 0.04) was observed in the left than the right side during early (0–5%) gait cycle of the TWT condition (Fig 4). The average increase in left knee flexion during that early phase of the gait cycle was 2.63±0.04° (Fig 4). Asymmetric ankle flexion-extension motions between sides were observed during the gait cycle in the GW and TWT conditions (Fig 5). Significant decreases in left ankle extension were 2.08±0.24° (*p* = 0.008) during the 48–58% of the gait cycle in the GW condition, and 1.6±0.1° (*p* = 0.05) during the 6–8% of the gait cycle in the TWT condition, respectively (Fig 5).

#### Asymmetric adduction-abduction motions

**Fig 6:**
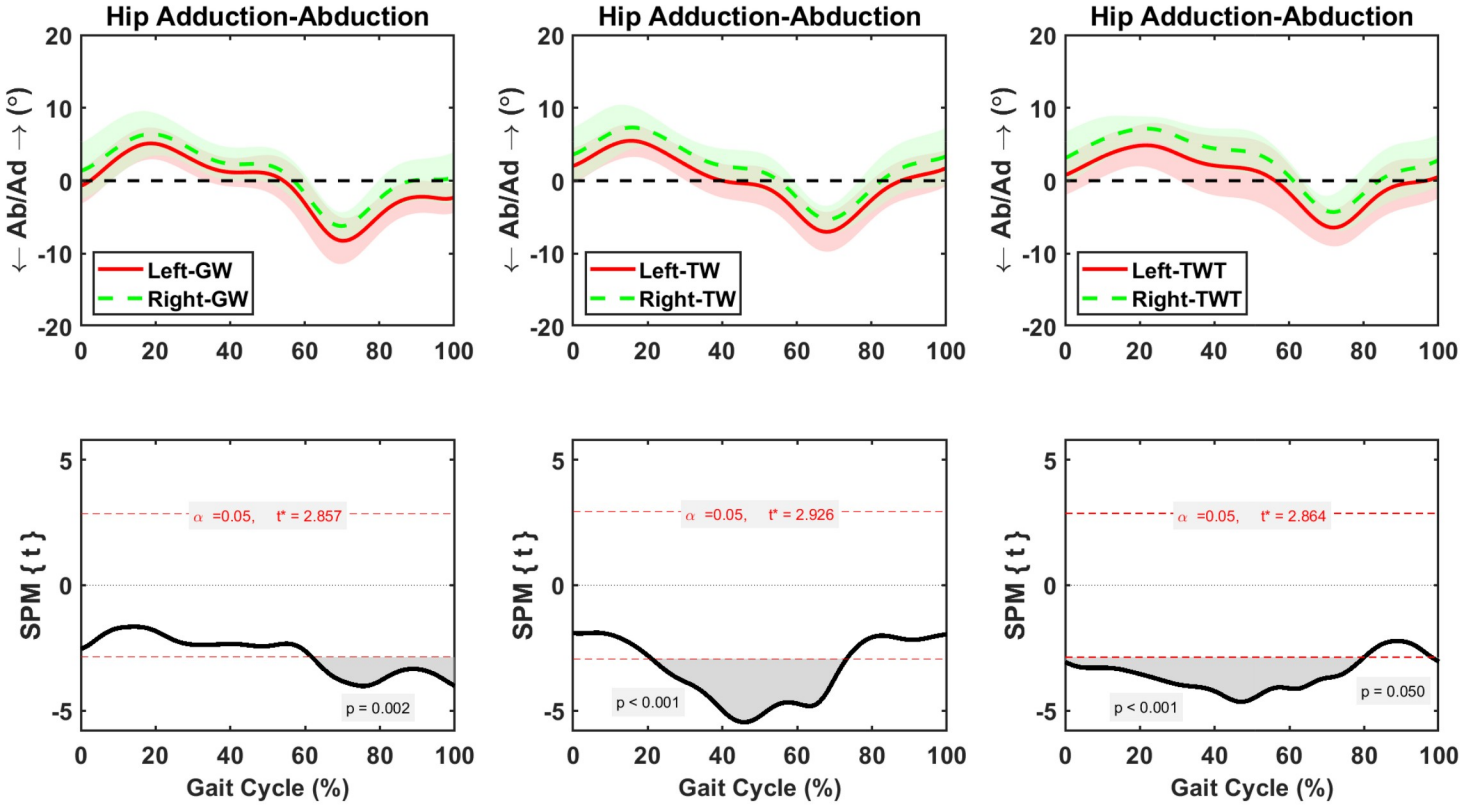
Average and standard deviation of hip abduction/ adduction (Ab/Ad) for left and right sides during one gait cycle of overground walking (GW), treadmill walking (TW), and treadmill walking while typing (TWT), in ten healthy participants. Scalar field SPM results with threshold at *t* >3.0 depicting where, in % cycle, left side angles were greater and lesser than right side angles. In upper panel: Solid and dashed lines correspond to average left and right sides, and shaded areas correspond to standard deviation.

**Fig 7:**
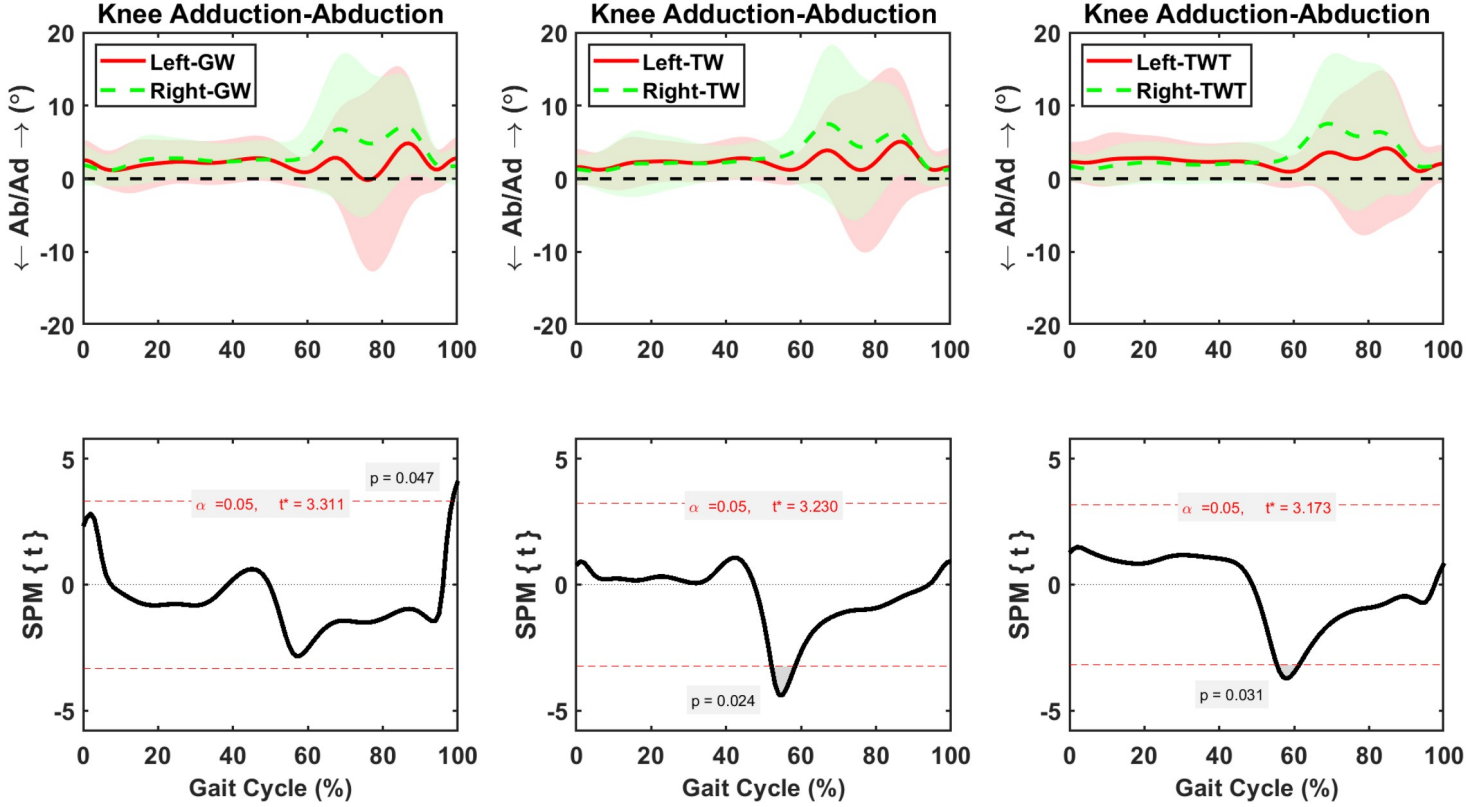
Average and standard deviation of knee abduction/ adduction (Ab/Ad) for left and right sides during one gait cycle of overground walking (GW), treadmill walking (TW), and treadmill walking while typing (TWT), in ten healthy participants. Scalar field SPM results with threshold at *t* >3.0 depicting where, in % cycle, left side angles were greater and lesser than right side angles. In upper panel: Solid and dashed lines correspond to average left and right sides, and shaded areas correspond to standard deviation.

**Fig 8:**
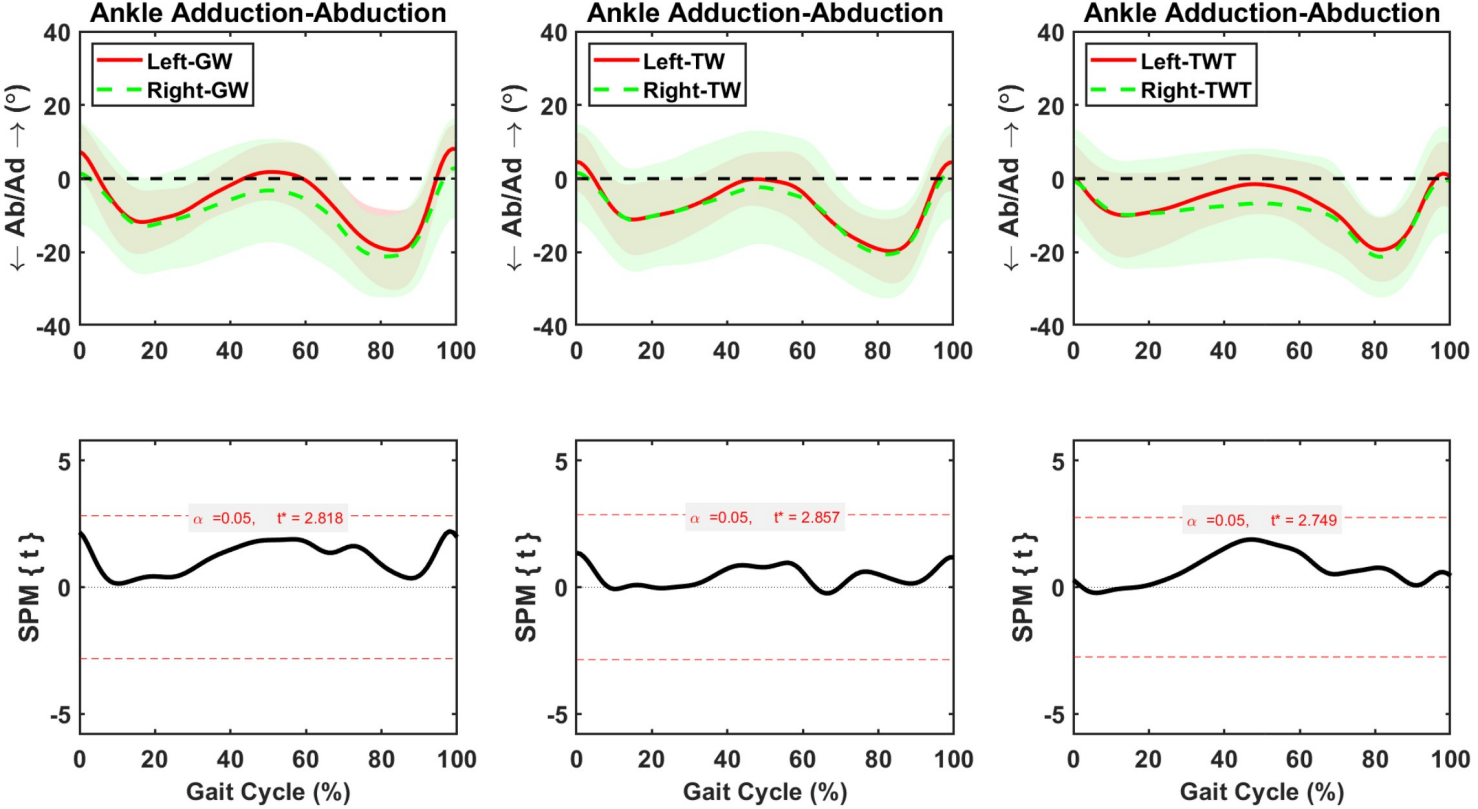
Average and standard deviation of ankle abduction/ adduction (Ab/Ad) for left and right sides during one gait cycle of overground walking (GW), treadmill walking (TW), and treadmill walking while typing (TWT), in ten healthy participants. Scalar field SPM results with threshold at *t* >3.0 depicting where, in % cycle, left side angles were greater and lesser than right side angles. In upper panel: Solid and dashed lines correspond to average left and right sides, and shaded areas correspond to standard deviation.

Hip adduction-abduction was asymmetric between sides during 62–100% of the GW, 21–73% of the TW, and 0–80% of TWT gait cycle, respectively (Fig 6). The average increases in right hip adduction were 2.2±0.3° (*p* = 0.002), during the 62–100% of the gait cycle in the GW condition, 1.94 ±0.1° (*p* < 0.001), during the 21–73% of the gait cycle in the TW condition, and 2.3±0.1° (*p* < 0.001), during the 0–80% of the gait cycle in the TWT condition, respectively (Fig 6). Knee adduction-abduction was asymmetric between left and right sides during short periods of GW (98–100%), TW (52–59%), and TWT (55–62%) gait cycles (Fig 7). The average changes in right knee adduction were 0.9±0.2° (*p* = 0.047), during the 98–100% of the gait cycle in the GW condition, 0.92±0.6° (*p* = 0.024), during the 52–59% of the gait cycle in the TW condition, and 1.67±0.65° (*p* = 0.031), during the 55–62% of the gait cycle in the TWT condition, respectively (Fig 7). Symmetric ankle adduction-abduction motions between sides were observed during the complete gait cycle of the GW, TW, and TWT (Fig 8).

## Discussion

This study investigated symmetry/asymmetry of lower limb motions during a typing task performed on a “walking-standing workstation.” Walking on the ground and on a treadmill, as well as typing while walking or standing on the treadmill were compared. Asymmetric lower limb motions were found between left and right hip, knee, and ankle joints during GW, TW, and TWT. The degree of interlimb asymmetry among conditions was comparable in all three conditions. This result was expected as asymmetrical behavior of the lower limbs during able-bodied ambulation was found to reflect natural functional differences between the lower extremities [38]. However, significantly asymmetric knee flexion-extension motions were only observed in treadmill workstation users in this cohort of participants. In fact, based on the average flexion-extension range of motion of the knee, the percentage of asymmetry during treadmill workstation use was about 4.5% compared to approximately 2% during overground and treadmill walking.

Self-selected walking speeds for overground and treadmill walking were similar to those reported previously [39,40,51], and walking speed differences between these two conditions were not generally significant, as already observed [52,53]. The walking speed was about 50% slower while typing (Fig 2) when compared to the other conditions. This concurs with other results obtained during active workstation use [54,55]. In addition, as we hypothesized, this result was expected due to the dual-task paradigm between walking and cognition. A common example that may be related to this phenomenon is the walking speed reduction by most people when they think deeply or interact with their cellphones [56].

Although some studies suggested that work performance is not affected by walking concurrently [54,55], others, suggested the opposite [34,35]. These apparent contradictions most likely stem from differences in the type of tasks performed and their respective cognitive requirements. In our study, typing speed, a cognitive performance measure, was reduced during treadmill walking. Walking while performing a computer typing task corresponds to a dual-task scenario that contributes to an increase in workload [32,36,37]. However, typing accuracy was not affected during treadmill walking. A possible account of this phenomenon could be that participants instinctively traded typing speed for typing accuracy, making accuracy their primary goal. Alternatively, since walking and typing speeds were reduced during workstation use, accuracy may become the primary goal by default. This hypothesis also concurs with the reversed perspective. Indeed, in young adults, walking does not interfere with low demand cognitive tasks, while more demanding task are altered [36,57]. Hence, the interaction between walking and another task is dependent on the task cognitive content since walking requires also some cognitive control [58,59].

Treadmill workstation may present physiological benefits over prolonged sitting such as increases in daily physical activity [18] and energy expenditure [19–21]. Moreover, the use of treadmill workstations may also help elude the adverse physiological effects of prolonged standing, since walking is shown to counteract long-lasting muscle fatigue [60,61]. However, the current study demonstrated knee flexion-extension asymmetries in treadmill workstation users. Workstation users presented increased knee flexion of the non-dominant leg (left leg) at early gait cycle. The lack of knee flexion-extension symmetry from workstation users was different from walking only conditions. Despite the fact that some degree of asymmetry within human gait may not have deleterious effects, the presence of considerably higher levels of asymmetry in workstation users could be problematic [62]. Significant movement asymmetries will overload one extremity, as it compensates for the diminished role of the contralateral extremity [38–40,62]. Over a long period of time, significant asymmetries may cause gait-related injuries, as seen in total hip and knee arthroplasty, as well as in post stroke patients [39–41,63–65]. As presented in Table 1, the degree of gait asymmetries reported in total hip and knee replacement studies was 8.6±4.6° for hip abduction-adduction [41], 2.5 ± 4.4° for knee abduction-adduction [39], and 4.3 ± 4.7° for knee flexion-extension [39] compared to 2.3±0.1° for hip abduction-adduction, 1.67±0.65° for knee abduction-adduction, and 2.63±0.04° for knee flexion extension during TWT. Therefore, asymmetries detected in treadmill workstation users were below the degree of asymmetries detected in total hip and knee replacement patients suggesting reduced risks associated with falls, discomfort, ailments, and potential injuries. However, our findings indicate that the average degree of asymmetry was greater during treadmill workstation use than during overground and treadmill walking (Table 1).

**Table 1:** Comparison of joint angle degree of asymmetry reported in clinical studies with our findings during walking.

*Hip Abduction-Adduction*			
THR	GW	TW	TWT
8.6±4.6° [41]	2.2±0.3°	1.9±0.1°	2.3±0.1°
*Knee Abduction-Adduction*			
TKR	GW	TW	TWT
2.5±4.4° [39]	0.9±0.2°	0.92±0.6°	1.67±0.65°
*Knee Flexion-Extension*			
TKR	GW	TW	TWT
4.3±4.7° [39]	1.2±0.1°^a^	0.4±0.1°^a^	2.6±0.04°

THR, total hip replacement; GW, overground walking; TW, treadmill walking; TWT, treadmill walking while typing; TKR, total knee replacement.

^a^ no significant difference was detected between left and right knees.

The present study demonstrated asymmetric lower body motions between left and right hip, knee, and ankle joints during GW, TW, and TWT in ten healthy participants. Various factors could contribute to the asymmetric lower body motions observed in this study. Walking on the ground does not require to control a constant walking speed as when walking on a treadmill, which demands greater attention [66] and thus higher requirement of visual control as gaze orientation is primarily accomplished by a coordination of eye and head movements in visually guided task and walking [67–70]. Human gait requires proper coordination of the lower extremity segments for optimal functioning [71,72]. However, even in healthy human gait, there are subtle asymmetries that exist to aid in the adaptation of changing walking environments [73]. Further research is needed to better understand gait and biomechanics adaptations associated with multitasking during treadmill workstation use. Similarly, as an ergonomic intervention, treadmill workstations are intended to be used for undefined time periods, as other office workstations. Therefore, long-term effects of treadmill workstation use on kinematics also needs to be investigated, especially for older office workers as walking control requires more attention with age [57,58] and gait asymmetry increases with age [73,74]. In addition, investigation of long-term effects of treadmill workstation use is required, as the compensatory patterns and resiliency of the human body may not be enough to maintain efficient pain-free function in the presence of long-term movement abnormalities such as misalignment, muscle imbalances [62], or severe gait asymmetries.

The results of the current study need to be interpreted in light of several limitations. First, the number of participants was relatively small, which would limit generalization. However, the sample size was projected based on a previous study describing the effects of walking workstations on biomechanical performance in nine participants [35], and differences between the tested conditions were statistically significant. Second, nine out of the ten participants were under 28 years old, and all participants reported a healthy lifestyle (exercised at least twice a week); hence, results may be limited to similar populations. Third, the cost (reduction of walking speed) of a single not too complex cognitive task was shown. However, it is not possible to determine what levels of cognitive load would correspond either to no interference between walking and performance or significant degradation of work performance while walking. Finally, few gait cycles with repetition were used in each condition; hence the long-term effects of walking while working were not explored.

## Conclusions

Despite comparable lower limb kinematic gait asymmetries during GW, TW, and TWT, asymmetric knee flexion-extension motions only persisted in treadmill workstation users. The results of the current study suggest that lower limb kinematic gait symmetry may be influenced by the use of treadmill workstations. The long-term effects (over a full day or multiple days of work) deserve attention to further understand gait symmetry adaptations and control, development of associated musculoskeletal disorders, and the likelihood of more severe interferences between working and walking in cognitive overload situations.
